# Evaluation of the Lower Limb Muscles’ Electromyographic Activity during the Leg Press Exercise and Its Variants: A Systematic Review

**DOI:** 10.3390/ijerph17134626

**Published:** 2020-06-27

**Authors:** Isabel Martín-Fuentes, José M. Oliva-Lozano, José M. Muyor

**Affiliations:** 1 Health Research Centre, University of Almería, 04120 Almería, Spain; imf902@ual.es (I.M.-F.); jol908@ual.es (J.M.O.-L.); 2 Laboratory of Kinesiology, Biomechanics and Ergonomics (KIBIOMER Lab.), Research Central Services, University of Almería, 04120 Almería, Spain

**Keywords:** electromyography, muscle activity, muscle activation, thigh muscles

## Abstract

The aim of this study was to analyze the literature on muscle activation measured by surface electromyography (sEMG) of the muscles recruited when performing the leg press exercise and its variants. The Preferred Reporting Items of Systematic Reviews and Meta-Analyses (PRISMA) guidelines were followed to report this review. The search was carried out using the PubMed, Scopus, and Web of Science electronic databases. The articles selected met the following inclusion criteria: (a) a cross-sectional or longitudinal study design; (b) neuromuscular activation assessed during the leg press exercise, or its variants; (c) muscle activation data collected using sEMG; and (d) study samples comprising healthy and trained participants. The main findings indicate that the leg press exercise elicited the greatest sEMG activity from the quadriceps muscle complex, which was shown to be greater as the knee flexion angle increased. In conclusion, (1) the vastus lateralis and vastus medialis elicited the greatest muscle activation during the leg press exercise, followed closely by the rectus femoris; (2) the biceps femoris and the gastrocnemius medialis showed greater muscular activity as the knee reached full extension, whereas the vastus lateralis and medialis, the rectus femoris, and the tibialis anterior showed a decreasing muscular activity pattern as the knee reached full extension; (3) evidence on the influence of kinematics modifications over sEMG during leg press variants is still not compelling as very few studies match their findings.

## 1. Introduction

The benefits of strength training [1] are strongly associated with different components of physical and mental health including reduced symptoms of depression, decreased lower back pain, and improved vascular condition, movement control and bone density as well as enhanced self-esteem, among others [2]. In addition, strength training can lead to significant differences in athletic performance by promoting greater force production, postural strength, ability to respond to stretch shortening cycles, and an increasing force development rate, which can result in faster muscle activation [3].

Although the selection of strength training exercises depends on the fitness goals (e.g., hypertrophy, performance, or injury prevention, among others), muscle activation patterns need to be considered. This is because greater muscle activation, and with it greater surface electromyographic (sEMG) amplitude and a greater number of motor units recruited, is normally essential for motor control, skills improvement, and strength and power development [4]. In humans, the force of a muscle contraction is partly regulated by the number, frequency, and synchronization of the motor units recruited [5,6]. Low threshold motor units are mainly recruited in low intensity exercises, whereas both low and high threshold motor units are recruited during high intensity exercises [6] or low intensity exercises performed to task failure [7].

Consequently, surface electromyography, which consists of a very sensitive voltmeter that detects increases and decreases in voltage on the sarcolemma, has become a common research tool in sports sciences for measuring muscle activation [8]. Accordingly, sEMG provides trainers and coaches with valuable information on electrical activity in muscles, in a simple and non-invasive way [9]. For example, sEMG amplitude and power spectral analysis are frequently used to examine muscle fatigue [10]. In this sense, an increase in the sEMG amplitude and a reduction in the sEMG spectral frequency manifest as the fatigue index increases [11,12].

Different studies have shown that muscle activation varies during multi-joint, lower-body strength training exercises depending on the intensity [6,12] and the variants of the exercise itself [12]. The leg press is performed using closed-chain kinetic effort [13] and the hip and knee extension involves large lower-body muscle groups (the quadriceps, hamstring, gluteus, and gastrocnemius) [14]. The specific training of these muscle groups is closely related to jumping, running, and athletic performance in general [15]. Consequently, the leg press exercise is widely used for strengthening the lower limbs [16,17,18,19].

Specifically, the leg press and its variants are some of the exercises studied in the scientific literature [14,15,20,21,22]. Previous studies have found that the knee flexion angle had a significant effect on muscle activation in the bilateral leg press [21,22]. For example, biceps femoris activation increases towards full knee extension [21,22], whereas the activation levels of the rectus femoris and vastus medialis decrease [22].

Additionally, certain variations in technique need to be considered when training using the leg press and its variants. For instance, feet position on the footplate, regarding lower and higher feet placement [14,15] or width stance [15] have been some studied variants of leg press. For example, leg press with high feet placement on the footplate showed higher gluteus maximus activity compared to low feet placement [14]. Conversely, low feet placement elicited greater rectus femoris activity compared to high feet placement [14]. On the contrary, Escamilla et al. (2001) [15] reported no significant differences on muscle activity between low and high feet placement.

With respect to feet rotation, the literature is also not compelling. In this regard, some authors attributed a preferential activation of vastus medialis to the external feet rotation [15,23], whereas others assessed the influence of knee external or internal resistance on vastus medialis during leg press [20,24]. Finally, various authors have assessed the influence of exercise intensity on muscle activity during the leg press exercises [6,12,25].

However, to the best of our knowledge, there is no comprehensive review of the literature on muscle activity during leg press exercises. The gathering of the current evidence is required to clarify which leg press exercise variant should be used to activate a specific targeted muscle.

The aim of this study, therefore, was to systematically review the current literature on muscle activity, measured by sEMG, of muscles recruited when performing the leg press exercise and all its best-known variants. It may provide athletes and coaches with a useful guide for lower limb strengthening during leg press exercises and its variants regardless of the training goals.

## 2. Materials and Methods

Search Protocol

The Preferred Reporting Items of Systematic Reviews and Meta-Analyses (PRISMA) [26] guidelines were followed to report this systematic review. A prior registration process was also conducted by the Cochrane Collaboration on the PROSPERO database. The quality of the studies was assessed by two independent reviewers using the PEDro quality scale; this comprised eleven questions, three of which were rejected due to their inability to blind trainees and researchers [27,28]. The protocol for this systematic review was registered on PROSPERO (CRD42020162417) and is available in full on the National Institute for Health Research’s site.

The literature search was performed using the PubMed, Scopus, and Web of Science electronic databases from December 2019 to January 2020, and included articles published from the databases’ inception up until December 2019.

The search strategy conducted on the different databases used the following related terms, Medical Subject Heading (MeSH) descriptors, and keywords: (a) PubMed: (“seated leg press” OR “leg press machine” OR “leg press” OR “angled leg press”) AND (“resistance training” OR “strength training” OR “resistance exercise” OR “weight lifting” OR “weight bearing”) AND (electromyography OR EMG OR “muscle activation” OR “muscle activity” OR “sEMG amplitude” OR “neuromuscular activation” OR “muscle excitation” OR “muscular activity”); (b) Scopus: (TITLE(“seated leg press” OR “leg press machine” OR “leg press” OR “angled leg press”) AND (“resistance training” OR “strength training” OR “resistance exercise” OR “weight lifting” OR “weight bearing”) AND (“electromyography” OR “EMG” OR “muscle activation” OR “muscle activity” OR “sEMG amplitude” OR “neuromuscular activation” OR “muscle excitation” OR “muscular activity”)); (c) Web of Science: ALL = ((“seated leg press”* OR “leg press machine”* OR “leg press”* OR “angled leg press”*) AND (“resistance training”* OR “strength training”* OR “resistance exercise”* OR “weight lifting”* OR “weight bearing”*) AND (electromyography* OR EMG* OR “muscle activation”* OR “muscle activity”* OR “sEMG amplitude”* OR “neuromuscular activation”* OR “muscle excitation”* OR “muscular activity”*)).

Each article included had to meet all the following inclusion criteria:

(1) a cross-sectional or longitudinal study design;
(2) neuromuscular activation assessed during the leg press exercise or its variants;
(3) EMG data collected using surface electromyography devices;
(4) study samples including healthy and trained participants older than eighteen years of age (with a minimum of six months resistance training experience).

Resistance training experience and familiarization with exercises may substantially alter the sEMG elicited during an exercise; for this reason, we only included studies with trained participants [2,29,30,31]. Studies that assessed upper-limb muscle activation and studies comparing the leg press exercise to any other lower-limb strengthening exercises were also considered. Although there were no language restrictions, all the studies selected were written in English. Publications such as abstracts, theses, books, book chapters, congress reviews, and articles that were not of minimum quality (according to the protocol description using the PEDro quality scale) were not considered.

Terminology such as “muscular activity”, “muscle excitation”, “neuromuscular activation” or “sEMG amplitude” have been used in the literature referring to electrical muscle activity. Due to the heterogeneity of terms used to describe the same concept, “muscle activation” is used for the current manuscript report.

**Figure 1:**
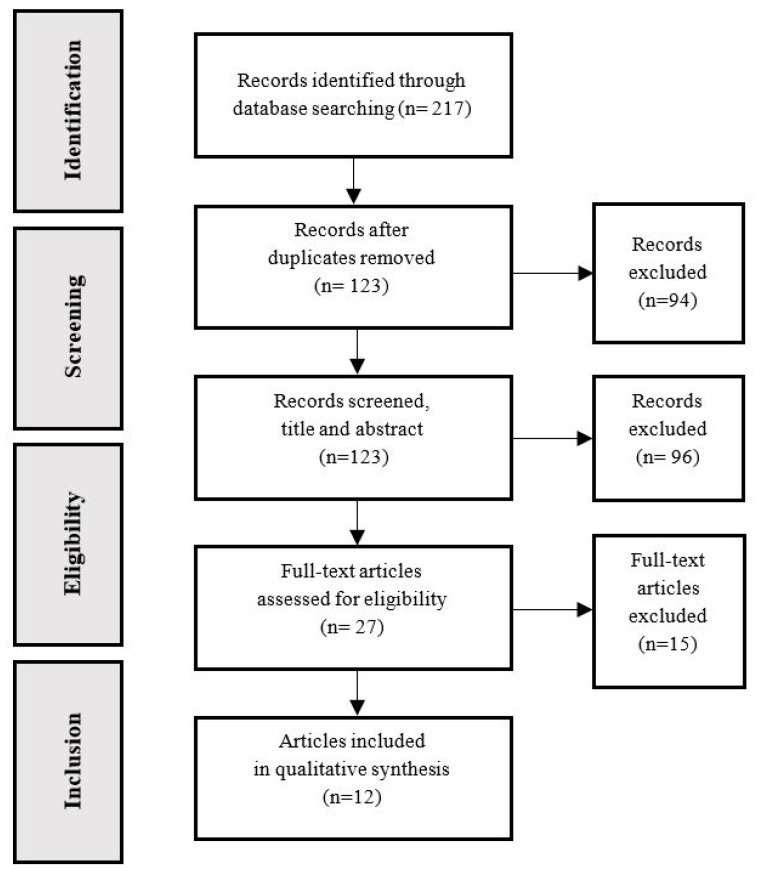
Flowchart.

Two independent reviewers selected the final articles to be included in the review in accordance with the inclusion and exclusion criteria. The first step consisted of filtering out duplicates. Next, the titles and abstracts were assessed, which led to a full text reading where necessary. In the case of disagreement between the two reviewers, a third reviewer was consulted. The search process lasted approximately three weeks. The identified articles were downloaded into the EndNote version X9 (Clarivate Analytics, New York, NY, USA) software for the subsequent data extraction process. All the steps followed are fully described in the flow chart (Figure 1).

The information extracted from the selected articles included the reference, the exercise movements measured, the sample size (n), the gender, the age (years), the experience (years), the muscles evaluated, the location of the electrodes, the limb tested (non-dominant/dominant), the sEMG collection method, the sEMG normalization method, the outcomes, and the main findings.

Finally, the selected studies reported muscle activation for every single exercise and muscle group separately. Thus, sEMG activity was the main data gathered and sEMG data on concentric and eccentric phases were collected where available.

It was not possible to perform a meta-analysis of the results because there was insufficient homogeneity of the methodological strategy and the type of analysis carried out in each study. For this reason, a qualitative results analysis was conducted for this review.

## 3. Results

### 3.1. Search Results

A total of 217 studies were identified from the initial database search. Ninety-four of them were immediately removed because they were duplicates, leading to a total of 123 records for the title and the abstract screening process. Twenty-seven studies were selected for full text reading; only twelve of these met the inclusion criteria and were eventually included in the review (Figure 1). Five studies were rejected directly because the samples included inexperienced participants. All the study participants reported a minimum of six months resistance training experience; this is because the experience time and exercise familiarization variable may significantly affect the muscle activation elicited during an exercise, hence the importance of having a homogeneous and experienced sample [30,31,32,33,34].

All the selected studies presented a cross-sectional study design with a randomized exercise testing order. Information regarding the studies’ general description are presented in Table 1. The selected studies were published between 2001 and December 2019. In addition, the final studies were categorized as being of good/excellent quality regarding the methodological and data reporting process, according to the PEDro quality scale.

The range of motion performed during the leg press exercises ranged from 120° to 0° knee flexion, with 0° being understood as a full knee extension, except for one study which interpreted 180° as the full knee extension [33]. The intensity performed during the exercises was diverse. In fact, some studies assessed a number of repetitions ranging between 30% and 90% of 1RM [6,12,14,16], some measured a number of maximum repetitions (xRM) [1,15,20], and others assessed the number of repetitions as x% of body weight (BW) [24,35]. In contrast, Hahn (2011) [22] performed the exercises at isometric positions.

No unified criteria were found regarding the sEMG normalization method. Although most studies used the % maximal voluntary isometric contraction (%MVIC) normalization method [1,12,15,20,22,24,33,34,35], other methods were used such as the % root mean square (%RMS) normalization method [14,20] and the average mean rectified voltage (MRV) [21], thus making it difficult to deliver consistent outcomes.

### 3.2. Muscles Assessed During Leg Press Exercises and Variants

In terms of sEMG activity, the vastus lateralis and the vastus medialis of the quadriceps are the muscles that have most been investigated during the leg press exercise and its variants (10/12 studies). The rectus femoris is the second most investigated muscle (8/12 studies), followed closely by the biceps femoris (7/12 studies). The muscles least investigated during leg press exercises include the gastrocnemius medialis (3/12 studies), and the gluteus maximus and the semitendinosus (1/12 studies each). Furthermore, only three studies reported muscle activation in the concentric and eccentric phase separately, and all of these identified greater muscle activation during the concentric phase of the exercise [1,20,21].

Due to the heterogeneity of the sEMG normalization methods, it was considered appropriate to report the results by grouping the studies into three categories: studies assessing muscle activation during the leg press exercise (Table 2), studies assessing muscle activation during other leg press variants (Table 3), and studies comparing the leg press with other exercises.

### 3.3. Leg Press Exercises

During the leg press, the horizontal movement ranges from a starting position of approximately 90° knee flexion to a final position with the knees totally extended (close to 0° knee flexion), performed while maintaining a slightly reclined seating position [22].

The quadriceps muscles, both the vastus medialis and the vastus lateralis, elicited the greatest muscle activation during the leg press, followed closely by the rectus femoris (Table 2). In addition, greater overall muscle activation was found for all the muscles as the leg press exercise intensity increased [6,12,33].

Regardless of the intensity applied during each of the five studies, the muscle activation pattern during the leg press exercise seemed to be similar for all of them (Table 2). The biceps femoris and the gastrocnemius medialis tended to show greater muscle activation as the knee extended to its full position [12,22,33]. On the other hand, different outcomes were found for the vastus lateralis, the vastus medialis, the rectus femoris of the quadriceps, and the tibialis anterior, showing a decrease in the muscle activation pattern as the knee reached its full extension [22,24,33].

Therefore, peak sEMG activity was found at approximately 90° knee flexion for the vastus lateralis, the vastus medialis, and the rectus femoris of the quadriceps, as well as for the tibialis anterior [22,24,33]. Furthermore, the biceps femoris and the gastrocnemius medialis presented their peak sEMG activity at an angle of 30° knee flexion [22,33]. Additionally, certain authors reported sEMG activity during single complete repetitions, without dividing the sEMG activity by the specific knee flexion angle [6,12].

### 3.4. Leg Press Variants

In addition to the leg press exercise itself, we found studies assessing several leg press variants. Some of them are the inclined leg press (45°) [1,14,20]; the leg press with extra hip adduction/abduction [20]; and the leg press with different feet height on the footplate (i.e., high and low), feet stances (i.e., narrow or wide), [15] and feet rotations on the footplate (0°–30° external rotation) [15]. The unilateral leg press [35] is, to the best of our knowledge, a leg press variant that has hardly been investigated (Table 1).

For instance, Escamilla et al. (2001) [15] assessed the leg press exercise using different feet stance positions, where leg press variants combining high and low, wide and narrow feet stances, and 0°or 30° feet external rotation were evaluated. The sEMG activity showed no significant differences among the different feet stances [15]. In line with these results, Da Silva et al. (2008) [14] also reported no differences using either high or low feet positions during the leg press. However, as exercise intensity increased to 80% 1RM, the gluteus maximus activity was greater when using the high feet position during the leg press [14].

An inclined leg press with an inclination angle of 45° (LP45) presented the greatest sEMG activity for the gluteus maximus [14] and for the vastus medialis of the quadriceps [20] in comparison to rectus femoris and biceps femoris [14,20].

When the leg press exercise was performed with extra hip adduction resistance (pressing a ball between the knees), greater hip adductor longus activity was reported [24]. There was also slightly greater vastus medialis activity reported during the leg press exercise with extra hip adduction resistance compared to the standard leg press [20]. Otherwise, leg press with hip abduction (elastic band around knees) significantly reduced the sEMG elicited by vastus medialis of the quadriceps in comparison to the standard leg press [20].

Finally, greater vastus medialis activity was found during the unilateral leg press exercise than during other unilateral weight-bearing exercises, such as the step-up, step-down, or straight leg raise [35].

## 4. Discussion

The main aim of the present study was to systematically review the current literature on muscle activation, measured with sEMG, of the muscles recruited when performing the leg press exercise and all its most known variants.

The relevant outcomes gathered from the literature revealed that the vastus medialis [1,6,12,15,20,21,22,24,33,35] and the vastus lateralis of the quadriceps [1,6,11,12,13,14,15,27] were the most studied muscles when performing the leg press exercise and its common variants, followed closely by the rectus femoris of the quadriceps muscles [1,6,12,14,15,20,22,33]. After this, the musculature activated most during leg press exercises were the muscles of the quadriceps complex, mainly the vastus medialis and the vastus lateralis. Indeed, this is the case for almost all the leg press variants studied [12,22,33].

Only three of the thirteen studies analyzed the sEMG data separately for each phase, reporting that concentric sEMG activity was greater than eccentric phase sEMG activity [1,20,21]. In the current literature, it has been widely stated that sEMG activity could significantly differ between the concentric and eccentric phase [36,37,38]. Thus, future studies using sEMG should seriously consider performing such subdivisions in their research.

### 4.1. Methodological Gaps Between the Studies

Diversity among the sEMG normalization methods was one of the most difficult issues to deal with in this review. Five different normalization methods were found among the selected studies (%MVIC, %RMS, MRV, RMS, and microvolts-µV), making it difficult to deliver consistent outcomes. Furthermore, the intensity at which exercises were performed was likewise diverse. Muscular activation is directly dependent on intensity, so wide differences in sEMG values could be seen when performing the same exercise but at different relative intensities [6,11,13,27]. Therefore, the intensity normalization method should be standardized when comparing several exercises from among the studies in order to achieve conclusive outcomes. For instance, apparently a ~70% 1RM intensity normalized as %MVIC might be set as an appropriate intensity for the strength training electromyographic studies [6,12,14,33,34]. Besides the disparities regarding the intensity and the sEMG normalization methods used in the studies included in the review, some other differences were found regarding the methodological process.

Even though the well-known Surface ElectroMyoGraphy for the Non-Invasive Assessment of Muscles (SENIAM) guidelines were followed in many studies [11,15,16,27], the criteria for electrode placement were varied. While certain studies did not even report any of the statements or guidelines followed [1,6,35], others followed the Cram guidelines [20,24], and some followed previously reported recommendations [14,15]. Nevertheless, surface electromyography data were collected during the same session for every study included in the review with the aim of avoiding electrode placement bias.

The sample sizes ranged between eight and eighteen subjects. Only three studies had a female sample [14,20,34], and just one evaluated a mixed sample [35]. More studies that include women are needed given the notable anthropometric and biomechanical differences between the sexes; muscle activation differences could also be found [39,40,41].

Finally, the inability of blinding trainees and researchers during measurements could be taken as a limitation for the data processing. However, this is an issue that is expected to have been controlled within every single study included into the review [27,28].

### 4.2. Leg Press Exercises

Studies gathered assessing sEMG during leg press agreed on the greatest sEMG activity elicited by vastus medialis and vastus lateralis in comparison to the rest of the muscles [6,12,33]. Although exercise intensity was in some studies slightly different, a greater overall sEMG muscle activity was observed for higher intensities compared to lower intensities [6,12,22,24,33]. As mentioned above, it is always advisable to consider exercise intensity during sEMG studies. Our findings provided useful information regarding sEMG muscle pattern during the leg press and peak sEMG activity of the muscles. Regardless of exercise intensity, vastus medialis, vastus lateralis, rectus femoris of the quadriceps, and tibialis anterior showed a decreased muscle activity as the knee extended to its full extension. Moreover, those muscles apparently showed their peak activity at an angle of 90° of the knee [22,24,33], whereas biceps femoris and gastrocnemius medialis increased their muscle activity as the knee extended, showing their peak muscle activity at an angle of 30° knee flexion [12,22,33]. This should be taken as key information in order to choose not only the best exercise for the individual case, but also the best range of motion when the aim is to target a specific muscle. For example, this knowledge is indispensable for injury prevention or return to play training programs, where ranges of motion are sometimes reduced or muscle activity must be controlled to ensure an appropriate recovery [41,42].

### 4.3. Leg Press Variants

Escamilla et al. (2001) [15] compared several leg press variants, and combined height feet placement (low and high), feet stance (wide and narrow), and feet abduction (0°–30°), concluding that the feet position did not affect thigh muscle activation in any of the tested variants. Thus, they recommended an individual self-selected comfortable feet position during the leg press exercise [15]. However, the intensity performed during sEMG data collection was very low (4 repetitions at 12RM intensity), which might have underestimated the findings [15]. More research should be conducted analyzing higher exercise intensities during the measurements.

On the other hand, some outcomes suggested that the muscle activation in the leg press with low foot placement (LPL) differed slightly from the muscle activation in the 45° inclined leg press. In this sense, the leg press with high foot placement (LPH) elicited greater gluteus maximus [14] and greater hamstring muscle activity [15] compared to the 45° inclined leg press exercise. Even though posterior thigh muscle activation is not the focus when performing leg press exercises, LPH could be performed, for instance, when one needs to decrease the intensity of the anterior thigh muscle activity during leg press exercise (e.g., for injury prevention) [35].

Furthermore, the leg press with extra hip adduction load (LP+/LP++) was found to be an interesting alternative when the target was to significantly increase medial thigh muscle activity, specifically hip adductor longus activation, by adding a ball between the knees to press [24]. Executing the leg press inclined at 45° with a physio ball between the knees (LP45BALL) also means increasing medial thigh muscle activity, in this case vastus medialis activation, compared to the standard leg press exercise [20]. Similarly, the unilateral leg press exercise showed great vastus medialis muscle activation. Although the unilateral leg press offers an interesting alternative to the standard leg press when the aim is to provide a functional perspective for the training program with transfer into daily activities (e.g., walking or climbing stairs) [35], one should consider the differences in absolute intensity that this unilateral exercise permits [35]. Overall, given that the literature on this topic remains scarce, outcomes need to be interpreted with caution.

### 4.4. Comparison of the Leg Press with Other Exercises

Although our aim did not cover exercises different from leg press and its variants, it would be interesting to outline some outcomes found within the studies included which compared leg press exercises and its variants to other exercises.

Bolgla et al. (2008) [35] concluded that the unilateral leg press elicited greater muscle activation for the vastus medialis of the quadriceps compared to other weight-bearing exercises such as the step-up, step-down, straight leg raise, air squat, or single leg stance. Consequently, the unilateral leg press could be the exercise of choice when targeting the vastus medialis during unilateral exercises.

In general terms, muscular activity increases as stability decreases. For instance, weight stack devices (e.g., the leg press machine) tend to facilitate exercise performance, which reduces the possibility of some synergist muscles taking part in the exercise [1,14]. Therefore, each exercise variant should be chosen based on the training goal. For example, different studies exposed leg press exercise as an optimal alternative within acute phases of rehabilitation and the return to play context in comparison to free weights, due to the movement stability [2,42,43].

We also found a study comparing the barbell front squat to several devices such as the flywheel leg press, the isokinetic knee extension dynamometer, the weight stack leg press, and the weight stack leg extension machine. The results showed that training using flywheel technology and isokinetic dynamometry provokes higher muscle activation (mainly during the eccentric phase of the movement) than traditional devices such as weight stack devices (the leg press) or barbells [1].

## 5. Conclusions

After performing the current systematic review, the following conclusions were reached:

(1) The vastus lateralis and vastus medialis of the quadriceps are the most investigated muscles in the leg press exercise and its variants (10/12 studies). The rectus femoris is the next most studied muscle (8/12 studies), followed closely by the biceps femoris (7/12 studies).
(2) The vastus medialis and vastus lateralis elicited the greatest muscle activation during the leg press exercise, followed closely by the rectus femoris.
(3) The biceps femoris and gastrocnemius medialis tended to show greater muscular activity as the knee extended to full extension, whereas the vastus lateralis, vastus medialis, rectus femoris of the quadriceps, and tibialis anterior showed a decrease in the muscular activity pattern as the knee reached its full extension.
(4) Greater muscle activation during the concentric phase of the exercise was found when these phases were electromyographically identified. This subdivision should be considered in future research.
(5) The participants’ training status and resistance training experience must be reported in detail.
(6) The sEMG normalization method, the intensity during data collection, the electrode placement, and the sample size and gender should all be standardized to obtain conclusive outcomes.
(7) It still remains unclear whether kinematics modifications such as knee external or internal resistance added, different feet stances, feet height, or feet rotations on the footplate may modify the sEMG elicited. More research is needed to investigate the different feet stances during the leg press exercise and its variants.

## 6. Practical Applications

The leg press is a well-known exercise performed in the fitness environment, used mainly to strengthen the lower limbs. It involves large lower-body muscle groups (the quadriceps, hamstring, gluteus, and gastrocnemius) and, as a closed-chain kinetic movement, it is closely related to jumping, running, and athletic performance in general.

Regardless of the leg press performed, the quadriceps muscles elicited the greatest muscle activation, principally the vastus medialis and vastus lateralis, followed closely by the rectus femoris. These muscles showed their greatest sEMG peak activity at a specific angle of 90° knee flexion, and the sEMG activity decreased as the knee reached its full extension. In contrast, the biceps femoris increased its sEMG activity progressively from 90° to 0° knee extension. This is key information in the injury prevention and return to play context, where the activation of target muscles at particular knee flexion degrees becomes indispensable information.

Regarding leg press variant exercises, findings are more controversial. Overall, results pointed out that the influence of the leg press kinematics modifications on the sEMG elicited is not fully clear. Although some studies cover some leg press variants, more research should be conducted in order to deliver consistent guidelines. However, we support the idea that a preferred self-selected feet stance should be encouraged, at least until there is strong evidence reporting a beneficial use of the so-called variants.

Furthermore, considering that several exercises currently exist that comprise large lower-body muscle groups, trainers, coaches and practitioners should also bear in mind that weight stack devices make it possible to train at high intensities while reducing the risk of injury to the athlete. Therefore, the leg press exercise and its variants could be exercises of choice for any strength training program, independent of the training goal.

## Figures and Tables

**Table 1:** General data regarding the exercises tested, the sample size, the participants’ gender and age, the training experience time, the surface electromyography (sEMG) collection method, the outcomes, and the main findings of the selected articles.

References	Exercises Tested	Sample	Age (Years)	Experience (Years)	sEMG Collection Method	sEMG Activity Recorded of Muscles	Main Findings
Escamilla et al. (2001) [15]	Squat versus leg press with different feet positions and stances (0° forefoot abduction, 30° forefoot abduction during wide and narrow stances. Leg press high and low feet position)	10 men Lifters	29.6 ± 6.5	10.1 ± 7.7 Squat, 9.0 ± 8.3 Leg press	4 reps 12RM	Biceps femoris, semitendinosus, vastus medialis, vastus lateralis, rectus femoris, and gastrocnemius	Foot abduction position did not affect thigh muscles’ activity during squat and leg press exercises.
Bolgla et al. (2008) [35]	Unilateral leg press versus step-up, step-down, straight leg raise, squat, single leg stance at 30° and full knee extension	8 women and 7 men	Women 22.2 ± 2.9, men 24.5 ± 3.2	Trained, not specified	3 reps 33% BW	Vastus medialis	Vastus medialis activity was greater during unilateral leg press than the rest of the exercises.
Da Silva et al. (2008) [14]	Leg press with low foot placement (LPL), high foot placement (LPH), and inclined to 45° (LP45)	14 women	21.5 ± 1.6	>6 months	5 reps 40%–80% 1RM	Gluteus maximus, biceps femoris, vastus lateralis, rectus femoris, and gastrocnemius	LPL and LP45 elicited greater rectus femoris and gastrocnemius activity at 40% and 80% 1RM. LPL elicited greater rectus femoris and vastus lateralis activity. LPH elicited greater gluteus maximus activity.
Gorostiaga et al. (2011) [21]	Leg press	13 men	34 ± 5	Trained, not specified	5–10reps at ~83% 1RM	Vastus medialis, vastus lateralis, and biceps femoris	Vastus medialis and vastus lateralis activity decreased progressively with extension. Biceps femoris activity was higher as extension increased.
Hahn (2011) [22]	Leg press at 8 distinct knee angles (30–100°)	18 men	30 ± 6.3	Trained, not specified	3 sets of 3 reps, 24 contractions per knee angle. Maximal isometric contraction	Biceps femoris, vastus medialis, rectus femoris, gastrocnemius medialis, and tibialis anterior	Vastus medialis and rectus femoris activity decreased with knee extension. Gluteus maximus and biceps femoris activity increased with knee extension. Tibialis anterior activity increased with knee flexion, peaking at 90–100° knee flexion.
Walker et al. (2011) [33]	Leg press at 2 s concentric phase and leg press with explosive concentric phase	9 men	29 ± 4.1	Trained, not specified	1 single rep per each technique. 40%–60%–80% 1RM	Biceps femoris, vastus medialis, vastus lateralis, and rectus femoris	Vastus medialis and vastus lateralis activity decreased progressively with extension. Biceps femoris activity remained low and consistent from 40°–120° knee flexion. No significant differences were observed, for any muscle, at any loading intensity, during explosive contractions.
Peng et al. (2013) [24]	Leg press versus leg press with submaximal isometric hip adduction force (LP+), and leg press with vigorous isometric hip adduct force (LP++)	10 men	21.0 ± 1.4	Trained, not specified	3 reps per exercise. 53 kg + 80% BW	Vastus medialis, vastus lateralis, and hip adductor longus	Greater hip adductor longus activity during LP++ for concentric and eccentric phase.
Schoenfeld et al. (2014) [12]	Leg press at 75% 1RM (high load) versus 30% 1RM (low load)	10 men	21.3 ± 1.5	Resistance trained >1 year	30% 1RM to 75% 1RM sets to failure	Biceps femoris, vastus medialis, vastus lateralis, and rectus femoris	Greater overall muscle activation during high load set. Greater vastus medialis and vastus lateralis activity than biceps femoris.
Gonzalez et al. (2017) [6]	Leg press to failure 70% 1RM and 90% 1RM	10 men	22.8 ± 2.7	4.6 ± 1.8 years	70%–90% 1RM reps to failure	Vastus medialis, vastus lateralis, and rectus femoris	Vastus lateralis elicited greater activity than rectus femoris, and rectus femoris elicited greater activity than vastus medialis. Greater overall muscle activation during 90% 1RM.
Machado et al. (2017) [20]	Leg press inclined 45° (LP45), LP45 with physio ball between knees, and LP45 with elastic band around knees	13 women	22.5 ± 2.9	Trained, not specified	10 reps 70% 10RM	Biceps femoris, vastus medialis, vastus lateralis, and rectus femoris	Greater vastus medialis activity during LP45 with physio ball between knees. Leg press with elastic band around knees increased rectus femoris activity.
Alkner and Bring (2019) [1]	Flywheel leg press, knee extension isokinetic dynamometry, barbell front squat, weight stack leg press, and weight stack knee extension	8 men	28 ± 6	Trained, not specified	8 reps 10RM	Vastus medialis, vastus lateralis, and rectus femoris	Flywheel technology and isokineticdynamometry induced higher eccentric muscle activation compared to traditional devices like barbells or weight stack devices.
Saeterbakken et al. (2019) [34]	Leg press, Smith machine and squat	19 women	24.1 ± 4.5	4.5 ± 2.0	3 reps 1RM	Rectus abdominis, oblique external, and erector spinae	Lower trunk muscle activation during leg press. Smith machine and squat elicited similar muscle activation.

Exercise abbreviations: LPH, high feet leg press; LPL, low feet leg press; LP45, 45° inclined leg press; LP+, leg press with isometric hip adduction; LP++, leg press with vigorous isometric hip adduction. Other abbreviations: BW, body weight; MVIC, maximal voluntary isometric contraction; reps, repetitions; RM, repetition maximum.

**Table 2:** Data on sEMG activity for studies assessing the leg press.

References	Exercise	Biceps Femoris	Vastus Medialis	Vastus Lateralis	Rectus Femoris	Gastrocnemius Medialis	Tibialis Anterior
Hahn (2011) [22]	Leg press 100°	~35% MVIC mean	~83% MVIC mean	n/a	~75% MVIC mean	~22% MVIC mean	~70% MVIC mean
	Leg press 90°	~40% MVIC mean	~80% MVIC mean	n/a	~73% MVIC mean	~26% MVIC mean	~77% MVIC mean
	Leg press 80°	~32% MVIC mean	~70% MVIC mean	n/a	~74% MVIC mean	~30% MVIC mean	~43% MVIC mean
	Leg press 70°	~32% MVIC mean	~75% MVIC mean	n/a	~76% MVIC mean	~40% MVIC mean	~22% MVIC mean
	Leg press 60°	~55% MVIC mean	~77% MVIC mean	n/a	~77% MVIC mean	~53% MVIC mean	~19% MVIC mean
	Leg press 50°	~78% MVIC mean	~69% MVIC mean	n/a	~60% MVIC mean	~69% MVIC mean	~18% MVIC mean
	Leg press 40°	~85% MVIC mean	~64% MVIC mean	n/a	~41% MVIC mean	~77% MVIC mean	~19% MVIC mean
	Leg press 30°	~83% MVIC mean	~50% MVIC mean	n/a	~23% MVIC mean	~89% MVIC mean	~17% MVIC mean
Walker et al. (2011) [33]	Leg press 40% 1RM 100–80°	~19% MVI mean	n/a	n/a	~55% MVIC mean	n/a	n/a
	Leg press 40% 1RM 80–60°	~19% MVIC mean	n/a	n/a	~31% MVIC mean	n/a	n/a
	Leg press 40% 1RM 60–40°	~22% MVIC mean	n/a	n/a	~18% MVIC mean	n/a	n/a
	Leg press 40% 1RM 40–20°	~25% MVIC mean	n/a	n/a	~9% MVIC mean	n/a	n/a
	Leg press 40% 1RM 20–0°	~37% MVIC mean	n/a	n/a	~6% MVIC mean	n/a	n/a
	Leg press 60% 1RM 100–80°	n/a	~127% MVIC mean	~120% MVIC mean	n/a	n/a	n/a
	Leg press 60% 1RM 80–60°	n/a	~115% MVIC mean	~105% MVIC mean	n/a	n/a	n/a
	Leg press 60% 1RM 60–40°	n/a	~98% MVIC mean	~100% MVIC mean	n/a	n/a	n/a
	Leg press 60% 1RM 40–20°	n/a	~75% MVIC mean	~80% MVIC mean	n/a	n/a	n/a
	Leg press 60% 1RM 20–0°	n/a	~69% MVIC mean	~45% MVIC mean	n/a	n/a	n/a
	Leg press 80% 1RM 100–80°	n/a	~150% MVIC mean	~148% MVIC mean	n/a	n/a	n/a
	Leg press 80% 1RM 80–60°	n/a	~125% MVIC mean	~120% MVIC mean	n/a	n/a	n/a
	Leg press 80% 1RM 60–40°	n/a	~110% MVIC mean	~120% MVIC mean	n/a	n/a	n/a
	Leg press 80% 1RM 40–20°	n/a	~100% MVIC mean	~98% MVIC mean	n/a	n/a	n/a
	Leg press 80% 1RM 20–0°	n/a	~90% MVIC mean	~65% MVIC mean	n/a	n/a	n/a
Peng et al. (2013) [24]	Leg press 90–75°	n/a	~50% MVIC mean	~55% MVIC mean	n/a	n/a	n/a
	Leg press 75–60°	n/a	~37% MVIC mean	~40% MVIC mean	n/a	n/a	n/a
	Leg press 60–45°	n/a	~33% MVIC mean	~37% MVIC mean	n/a	n/a	n/a
	Leg press 45–30°	n/a	~23% MVIC mean	~29% MVIC mean	n/a	n/a	n/a
	Leg press 30–15°	n/a	~19% MVIC mean	~23% MVIC mean	n/a	n/a	n/a
	Leg press 15–0°	n/a	~15% MVIC mean	~21% MVIC mean	n/a	n/a	n/a
Schoenfeld et al. (2014) [12]	Leg press 30% 1RM	~19% MVIC peak	~74% MVIC peak	~70% MVIC peak	n/a	n/a	n/a
	Leg press 75% 1RM	~72% MVIC peak	~210% MVIC peak	~195% MVIC peak	n/a	n/a	n/a
Gonzalez et al. (2017) [6]	Leg press 70% 1RM	n/a	~60% MVIC mean	~75% MVIC mean	~59% MVIC mean	n/a	n/a
	Leg press 90% 1RM	n/a	~65% MVIC mean	~79% MVIC mean	~68% MVIC mean	n/a	n/a

Abbreviations: MVIC, maximal voluntary isometric contraction; RM, repetition maximum; n/a, not available.

**Table 3:** Data on sEMG activity in studies comparing the leg press with some of its variants.

References	Exercise	Medial Hamstrings	Lateral Hamstrings	Biceps Femoris	Vastus Medialis	Vastus Lateralis	Rectus Femoris	Gastrocnemius Medialis	Gluteus Maximus	Hip Adductor Longus
Escamilla et al. (2001) [15]	Leg press high feet narrow stance	~15% MVIC peak	~13% MVIC peak	n/a	n/a	~47% MVIC peak	~39% MVIC peak	~14% MVIC peak	n/a	n/a
	Leg press high feet wide stance	~20% MVIC peak	~16% MVIC peak	n/a	n/a	~50% MVIC peak	~33% MVIC peak	~15% MVIC peak	n/a	n/a
	Leg press low feet narrow stance	~11% MVIC peak	~12% MVIC peak	n/a	n/a	~48% MVIC peak	~46% MVIC peak	~22% MVIC peak	n/a	n/a
	Leg press low feet wide stance	~15% MVIC peak	~12% MVIC peak	n/a	n/a	~50% MVIC peak	~37% MVIC peak	~22% MVIC peak	n/a	n/a
Bolgla et al. (2008) [35]	Unilateral leg press	n/a	n/a	n/a	41% ± 19% MVIC mean	n/a	n/a	n/a	n/a	n/a
Da Silva et al. (2008) [14]	Leg press low feet 40% 1RM	n/a	n/a	~42% RMS	n/a	~50% RMS	~38% RMS	~26% RMS	~40% RMS	n/a
	Leg press high feet 40% 1RM	n/a	n/a	~44% RMS	n/a	~50% RMS	~27% RMS	~38% RMS	~37% RMS	n/a
	Leg press inclined 45° 40% 1RM	n/a	n/a	~46% RMS	n/a	~52% RMS	~48% RMS	~40% RMS	~39% RMS	n/a
	Leg press low feet 80% 1RM	n/a	n/a	~96% RMS	n/a	~95% RMS	~96% RMS	~77% RMS	~81% RMS	n/a
	Leg press high feet 80% 1RM	n/a	n/a	~85% RMS	n/a	~81% RMS	~63% RMS	~40% RMS	~115% RMS	n/a
	Leg press inclined 45° 80% 1RM	n/a	n/a	~81% RMS	n/a	~87% RMS	~87% RMS	~74% RMS	~100% RMS	n/a
Peng et al. (2013) [24]	Leg press	n/a	n/a	n/a	25.53% ± 8.43% MVIC mean	31.28% ± 11.83% MVIC mean	n/a	n/a	n/a	7.44% ± 4.69% MVIC mean
	Leg press+	n/a	n/a	n/a	26.53% ± 10.06% MVIC mean	32.36% ± 12.97% MVIC mean	n/a	n/a	n/a	11.12% ± 6.55% MVIC mean
	Leg press++	n/a	n/a	n/a	26.77% ± 10.21% MVIC mean	33.84% ± 14.91% MVIC mean	n/a	n/a	n/a	20.83% ± 8.58% MVIC mean
Machado et al. (2017) [20]	Leg press inclined 45°	n/a	n/a	~17% RMS	~80% RMS	~70% RMS	~42% RMS	n/a	n/a	n/a
	Leg press 45° with physio ball between knees	n/a	n/a	~18% RMS	~100% RMS	~98% RMS	~39% RMS	n/a	n/a	n/a
	Leg press inclined 45° with elastic band around knees	n/a	n/a	~19% RMS	~43% RMS	~76% RMS	~63% RMS	n/a	n/a	n/a

Abbreviations: +, leg press with isometric hip adduction; ++, leg press with vigorous isometric hip adduction; MVIC, maximal voluntary isometric contraction; RM, repetition maximum; RMS, root mean square; n/a, not available.

## References

[1] Alkner B.A., Bring D.K. (2019). Muscle activation during gravity-independent resistance exercise compared to common exercises. Aerosp. Med. Hum. Perform..

[2] Westcott W.L. (2012). Resistance training is medicine. Curr. Sports Med. Rep..

[3] DeWeese B.H., Hornsby G., Stone M., Stone M.H. (2015). The training process: Planning for strength–power training in track and field. Part 1: Theoretical aspects. J. Sport Health Sci..

[4] Schoenfeld B.J., Peterson M.D., Ogborn D., Contreras B., Sonmez G.T. (2015). Effects of low vs. high load resistance training on muscle strength and hypertrophy in well-trained men. J. Strength Cond. Res..

[5] Fuglsang-Frederiksen A., Rønager J. (1988). The motor unit firing rate and the power spectrum of EMG in humans. Electroencephalogr. Clin. Neurophysiol..

[6] Gonzalez A.M., Ghigiarelli J.J., Sell K.M., Shone E.W., Kelly C.F., Mangine G.T. (2017). Muscle activation during resistance exercise at 70% and 90% 1-repetition maximum in resistance-trained men. Muscle Nerve.

[7] Morton R.W., Sonne M.W., Farias Zuniga A., Mohammad I.Y.Z., Jones A., McGlory C., Keir P.J., Potvin J.R., Phillips S.M. (2019). Muscle fibre activation is unaffected by load and repetition duration when resistance exercise is performed to task failure. J. Physiol..

[8] Stegeman D., Hermens H.J. (2007). Standards for suface electromyography: The European project surface EMG for non-invasive assessment of muscles (SENIAM). Enschede Roessingh Res. Dev..

[9] Vigotsky A.D., Halperin I., Lehman G.J., Trajano G.S., Vieira T.M. (2018). Interpreting signal amplitudes in surface electromyography studies in sport and rehabilitation sciences. Front. Physiol..

[10] Farina D., Merletti R., Enoka R.M. (2004). The extraction of neural strategies from the surface EMG. J. Appl. Physiol..

[11] Hunter S.K., Duchateau J., Enoka R.M. (2004). Muscle fatigue and the mechanisms of task failure. Exerc. Sport Sci. Rev..

[12] Schoenfeld B.J., Contreras B., Willardson J.M., Fontana F., Tiryaki-Sonmez G. (2014). Muscle activation during low versus high load resistance training in well-trained men. Eur. J. Appl. Physiol..

[13] Ivey F.M., Prior S.J., Hafer-Macko C.E., Katzel L.I., Macko R.F., Ryan A.S. (2017). Strength training for skeletal muscle endurance after stroke. J. Stroke Cerebrovasc. Dis..

[14] Da Silva E.M., Brentano M.A., Cadore E.L., De Almeida A.P.V., Kruel L.F.M. (2008). Analysis of muscle activation during different leg press exercises at submaximum effort levels. J. Strength Cond. Res..

[15] Escamilla R.F., Fleisig G.S., Zheng N., Lander J.E., Barrentine S.W., Andrews J.R., Bergemann B.W., Moorman C.T. (2001). Effects of technique variations on knee biomechanics during the squat and leg press. Med. Sci. Sports Exerc..

[16] Rossi F.E., Schoenfeld B.J., Ocetnik S., Young J., Vigotsky A., Contreras B., Krieger J.W., Miller M.G., Cholewa J. (2018). Strength, body composition, and functional outcomes in the squat versus leg press exercises. J. Sports Med. Phys. Fitness.

[17] da R. Orssatto L.B., de Moura B.M., Sakugawa R.L., Radaelli R., Diefenthaeler F. (2018). Leg press exercise can reduce functional hamstring: Quadriceps ratio in the elderly. J. Bodyw. Mov. Ther..

[18] Monteiro E.R., Steele J., Novaes J.S., Brown A.F., Cavanaugh M.T., Vingren J.L., Behm D.G. (2019). Men exhibit greater fatigue resistance than women in alternated bench press and leg press exercises. J. Sports Med. Phys. Fitness.

[19] Harden M., Wolf A., Russell M., Hicks K.M., French D., Howatson G. (2018). An evaluation of supramaximally loaded eccentric leg press exercise. J. Strength Cond. Res..

[20] Machado W., Paz G., Mendes L., Maia M., Winchester J.B., Lima V., Willardson J.M., Miranda H. (2017). Myoeletric activity of the quadriceps during leg press exercise performed with differing techniques. J. Strength Cond. Res..

[21] Gorostiaga E.M., Navarro-Amezqueta I., Gonzalez-Izal M., Malanda A., Granados C., Ibanez J., Setuain I., Izquierdo M. (2012). Blood lactate and sEMG at different knee angles during fatiguing leg press exercise. Eur. J. Appl. Physiol..

[22] Hahn D. (2011). Lower extremity extension force and electromyography properties as a function of knee angle and their relation to joint torques: Implications for strength diagnostics. J. Strength Cond. Res..

[23] Smith T.O., Bowyer D., Dixon J., Stephenson R., Chester R., Donell S.T. (2009). Can vastus medialis oblique be preferentially activated? A systematic review of electromyographic studies. Physiother. Theory Pract..

[24] Peng H.-T., Kernozek T.W., Song C.-Y. (2013). Muscle activation of vastus medialis obliquus and vastus lateralis during a dynamic leg press exercise with and without isometric hip adduction. Phys. Ther. Sport.

[25] Clark D.R., Lambert M.I., Hunter A.M. (2017). Trunk muscle activation in the back and hack squat at the same relative loads. J. Strength Cond. Res..

[26] Moher D., Liberati A., Tetzlaff J., Altman D.G. (2009). Prisma Group Preferred reporting items for systematic reviews and meta-analyses: The PRISMA statement. J. Clin. Epidemiol..

[27] Neto W.K., Vieira T.L., Gama E.F. (2019). Barbell hip thrust, muscular activation and performance: A systematic review. J. Sports Sci. Med..

[28] Martín-Fuentes I., Oliva-Lozano J.M., Muyor J.M. (2020). Electromyographic activity in deadlift exercise and its variants. A systematic review. PLoS ONE.

[29] Westcott W., Winett R., Annesi J., Wojcik J., Anderson E., Madden P. (2009). Prescribing physical activity: Applying the ACSM protocols for exercise. Physician Sportsmed..

[30] Calatayud J., Vinstrup J., Jakobsen M.D., Sundstrup E., Colado J.C., Andersen L.L. (2017). Mind-muscle connection training principle: Influence of muscle strength and training experience during a pushing movement. Eur. J. Appl. Physiol..

[31] Rutherford O.M., Jones D.A. (1986). The role of learning and coordination in strength training. Eur. J. Appl. Physiol. Occup. Physiol..

[32] Wulf G. (2013). Attentional focus and motor learning: A review of 15 years. Int. Rev. Sport Exerc. Psychol..

[33] Walker S., Peltonen H., Avela J., Hakkinen K. (2011). Kinetic and electromyographic analysis of single repetition constant and variable resistance leg press actions. J. Electromyogr. Kinesiol..

[34] Saeterbakken A.H., Stien N., Pedersen H., Andersen V. (2019). Core muscle activation in three lower extremity with different stability requirements. J. Strength Cond. Res..

[35] Bolgla L.A., Shaffer S.W., Malone T.R. (2008). Vastus medialis activation during knee extension exercises: Evidence for exercise prescription. J. Sport Rehabil..

[36] Ono T., Okuwaki T., Fukubayashi T. (2010). Differences in activation patterns of knee flexor muscles during concentric and eccentric exercises. Res. Sports Med..

[37] Matheson J.W., Kernozek T.W., Fater D.C., Davies G.J. (2001). Electromyographic activity and applied load during seated quadriceps exercises. Med. Sci. Sports Exerc..

[38] Komi P.V., Linnamo V., Silventoinen P., Sillanpää M. (2000). Force and EMG power spectrum during eccentric and concentric actions. Med. Sci. Sports Exerc..

[39] Bouillon L.E., Wilhelm J., Eisel P., Wiesner J., Rachow M., Hatteberg L. (2012). Electromyographic assessment of muscle activity between genders during unilateral weight-bearing tasks using adjusted distances. Int. J. Sports Phys. Ther..

[40] Schwanbeck S.R., Cornish S., Barss T., Chilibeck P.D. (2020). Effects of training with free weights versus machines on muscle mass, strength, free testosterone and free cortisol levels. J. Strength Cond. Res..

[41] Khaiyat O.A., Norris J. (2018). Electromyographic activity of selected trunk, core, and thigh muscles in commonly used exercises for ACL rehabilitation. J. Phys. Ther. Sci..

[42] Trevino M.A., Herda T.J. (2015). The effects of training status and muscle action on muscle activation of the vastus lateralis. Acta Bioeng. Biomech..

[43] El-Ashker S., Chaabene H., Prieske O., Abdelkafy A., Ahmed M.A., Muaidi Q.I., Granacher U. (2019). Effects of neuromuscular fatigue on eccentric strength and electromechanical delay of the knee flexors: The role of training status. Front. Physiol..

